# A Network-Based Approach to Investigate the Pattern of Syndrome in Depression

**DOI:** 10.1155/2015/768249

**Published:** 2015-03-02

**Authors:** Jianglong Song, Xi Liu, Qingqiong Deng, Wen Dai, Yibo Gao, Lin Chen, Yunling Zhang, Jialing Wang, Miao Yu, Peng Lu, Rongjuan Guo

**Affiliations:** ^1^Institute of Automation, Chinese Academy of Sciences, Beijing 100190, China; ^2^College of Information Science and Technology, Beijing Normal University, Beijing 100875, China; ^3^Dongfang Hospital, Beijing University of Chinese Medicine, Beijing 100029, China

## Abstract

In Traditional Chinese Medicine theory, syndrome is essential to diagnose diseases and treat patients, and symptom is the foundation of syndrome differentiation. Thus the combination and interaction between symptoms represent the pattern of syndrome at phenotypic level, which can be modeled and analyzed using complex network. At first, we collected inquiry information of 364 depression patients from 2007 to 2009. Next, we learned classification models for 7 syndromes in depression using naïve Bayes, Bayes network, support vector machine (SVM), and C4.5. Among them, SVM achieves the highest accuracies larger than 0.9 except for Yin deficiency. Besides, Bayes network outperforms naïve Bayes for all 7 syndromes. Then key symptoms for each syndrome were selected using Fisher's score. Based on these key symptoms, symptom networks for 7 syndromes as well as a global network for depression were constructed through weighted mutual information. Finally, we employed permutation test to discover dynamic symptom interactions, in order to investigate the difference between syndromes from the perspective of symptom network. As a result, significant dynamic interactions were quite different for 7 syndromes. Therefore, symptom networks could facilitate our understanding of the pattern of syndrome and further the improvement of syndrome differentiation in depression.

## 1. Introduction

In Traditional Chinese Medicine (TCM) theory, syndrome, also known as ZHENG, is a primary and essential concept to diagnose disorders and treat patients [1]. Different from western medicine, syndrome is the criterion to distinguish disease states and the basis to determine TCM therapies. In the procedure of diagnosis and treatment, TCM doctors always judge which syndromes a patient belongs to through inspection, auscultation and olfaction, inquiry, and palpation (four diagnostic methods of TCM); then they give medical care to the patient using acupuncture and/or TCM herbal formulae according to previous diagnosis. Thus, syndrome differentiation is of vital importance in TCM research and clinical practice [2, 3]. However, due to the subjectivity and complexity of syndromes, it is a challenging task to correctly discriminate the syndrome that a patient actually suffers from [1, 2].

Although syndromatology along with TCM has developed for more than 3000 years, there are still several inevitable disadvantages that block the improvement of syndrome differentiation. With TCM knowledge accumulated for thousands of years, classic techniques to identify syndrome include observation, listening, questioning, and pulse analysis [2], which mostly depend on empirical opinions of TCM doctors and subjective feelings of patients. Hence, such inevitable characteristics block the process of TCM modernization to drive TCM into an evidence-base medicine [4]. For example, when a patient tells a TCM doctor how he feels, what he describes can hardly be measured quantitatively; when a doctor differentiates the syndromes a patient may belong to, the diagnosis is made based on the doctor's experience and may not acquire approval of other TCM doctors. One common solution is to roughly quantify the level of each symptom using discrete numerals and represent the existence of certain syndrome by binary values. Plenty of work in syndrome differentiation has been accomplished using data sets generated by this technique [5–7]. In addition, some precise instruments and accurate models were also employed in syndrome differentiation in recent years [8, 9]. Up to now, it becomes popular to predict syndrome using classical machine learning models on the basis of scaled symptom data [5, 10, 11]. In fact, such classification models always exhibit excellent performance, facilitating the development of computer-aided syndrome differentiation.

Although lots of classification models could achieve high accuracy in syndrome differentiation, it is still a difficult task to uncover and understand the pattern of syndrome at symptomatic level. Typical methods including feature selection usually discover a set of symptoms highly related to a syndrome under study [6, 12]. However, the interaction between symptoms for a given syndrome has not been explored by such methods. As a matter of fact, the interacting profile of symptoms has informative impact on syndrome differentiation from a practical perspective. Fortunately, network pharmacology provides new insight into TCM modernization as well as the research of syndrome classification [13, 14]. Li proposed a novel construct, that is, to investigate syndrome based on biological molecular networks and phenotype combination [15, 16]. In spite of interpreting syndrome at gene/protein level, plenty of work focused on the improvement of syndrome classification using phenotypic network [10] or symptom interaction network [17]. Moreover, Zhou and Liu established a network analysis system for TCM clinical data, which involved herb network, symptom network, diagnosis network, and so on [18]. Therefore, together with the classification model, network analysis of symptoms holds great promise to uncover the pattern of syndrome in certain disease and further facilitate TCM diagnosis and treatment.

In this paper, we firstly learned classification models for syndrome differentiation in depression using four different algorithms, including naïve Bayes, Bayes network, SVM, and C4.5. Then we selected key symptoms related to each syndrome in depression using Fisher's score. Using top 12 key symptoms, 7 symptom networks were constructed for 7 syndromes and a global network for depression using all symptoms. We analyzed the topological properties of symptom networks from a global perspective. Then, through permutation test, dynamic interactions were discovered from symptom networks for 7 syndromes. So the dynamic interactions together with key symptoms uncovered the pattern of syndromes in depression.

## 2. Materials and Methods

### 2.1. Depression Dataset

From 2007 to 2009, we collected inquiry information of 364 patients who had been diagnosed with depression. Our work was approved by the Dongfang Hospital Ethics Committee of Beijing University of Chinese Medicine, and every patient signed an informed consent that explained the usage of their clinical information. The inquiry information was primarily comprised of risk degree of patients in terms of 36 symptoms including two specified to females. Each symptom defines a kind of typical physical signs as described in Table 1. Given a patient, the risk degree of each symptom was estimated by four grades: none (0), low (1), median (2), and high (3). Besides, TCM experts differentiated syndromes for every patient with their rich experience. There were 7 syndromes studied in this dataset of depression, which were blood deficiency (BD), blood stasis (BS), fire hot (FH), phlegmatic hygrosis (PH), Qi deficiency (QD), Qi stagnation (QS), and Yin deficiency (YD). Each case was labeled by 1 or 0 to indicate whether a patient belonged to some syndrome or not. So we acquired a scaled dataset of depression for further experiments and analysis.

### 2.2. Classification Test

Based on the roughly quantified dataset, we could learn classification models to differentiate syndromes in depression. Instead of training single model of multiple-label assignment as conventional ways, we generated models for each of 7 syndromes under study. To learn an accurate and effective model for each syndrome, 4 different methods were employed to train models and make predictions. These 4 classic methods are naïve Bayes (NB), Bayes network (BN), support vector machine (SVM), and C4.5.

A naïve Bayes classifier is essentially a simplified version of Bayes network. Slightly different from common Bayes network, a naïve Bayes classifier is a probabilistic model based on strong independence assumption that all features are conditionally independent of each other, given a class variable. Although this assumption goes against real-world situation in most cases, naïve Bayes classifiers always maintain good performance in two-class problem. Assuming that there are *k* features {*F*_1_, *F*_2_,…, *F*_*k*_} and one class variable *C*, the classifier derived from the MAP (*maximum a posteriori*) decision rule of Bayes probability model previously trained can be expressed as follows:

(1) $$\begin{array}{c} C_{f_{1},\ldots ,f_{k}}=\arg\underset{c}{\max}\;P\left(c\;|\;f_{1},\ldots ,f_{k}\right), \end{array}$$

(2) $$\begin{array}{c} P\left(C\;|\;F_{1},\ldots ,F_{k}\right)=\alpha \cdot P\left(C\right)\overset{k}{\underset{i=1}{\prod }}P\left(F_{i}\;|\;C\right), \end{array}$$

where *α* = *P*(*F*_1_,…, *F*_*k*_) is a normalization constant and makes no contribution to the classification. According to (1), a sample with feature vector (*f*_1_,…, *f*_*k*_) will be classified into a category which maximizes the posterior probability *P*(*c*∣*f*_1_,…, *f*_*k*_).

A Bayes network is a probabilistic graphical model that represents a set of random variables and their conditional dependencies via a directed acyclic graph (DAG). In a DAG of Bayes network, the nodes are feature variables and class variable, and the directed links represent conditional dependencies among these variables. Besides, there is a set of parameters quantifying the DAG, which is in fact a group of conditional probabilities. Thus, through the process of structure learning and parameter learning, a Bayes network is eventually constructed to predict the class of new instances. The Bayes network can be represented by the following function:

(3) $$\begin{array}{c} P_{G}\left(C\;|\;F_{1},\ldots ,F_{k}\right)=\alpha \cdot P_{G}\left(C\right)\overset{k}{\underset{i=1}{\prod }}P_{G}\left(F_{i}\;|\;{\text{Pa}}_{i}\right), \end{array}$$

where *G* is the DAG of Bayes network; Pa_*i*_ is the parent set of feature *F*_*i*_ in *G*. However, due to the complexity and diversity of dependent relations between variables, it is always a difficult task to learn an exact DAG fitting the training data. Here, we adopted a relatively simple strategy, tree augmented naïve Bayes (TAN), to learn model based on the training set [19]. The structure and parameters of TAN were learned by building a minimum weighted spanning tree involving all feature and class variables.

A support vector machine (SVM) is one of the most important supervised learning models to analyze medical data and recognize disease patterns [11, 20]. A SVM takes a set of input samples represented by *k*-dimensional vectors and learns a (*k* − 1)-dimensional hyperplane as decision surface which maximizes the margin between two classes. The learning of SVM model can be transformed into the optimization of quadratic programming problem. Moreover, the conventional SVM was developed by combining slack variable and using kernel trick. The former makes SVM become a soft margin method that is able to balance a large margin with a small error rate. The latter surprisingly introduces SVM to nonlinear classification by using different kinds of nonlinear kernel functions which implicitly map the input into high-dimensional feature space. Currently, there are four types of widely used kernel functions: linear function, polynomial function, radial basis function, and sigmoid function. In this paper, we applied C-support vector classifier with linear kernel function to learn models for syndrome classification.

C4.5 is an excellent algorithm used to generate a decision tree for classification. According to the procedure of C4.5 algorithm, the feature with the highest normalized information gain is selected to split the training set apart, and then similar process is recursively applied to the subsets of last step. After the tree is initially constructed, a pruning process is performed to eliminate undesired subtrees. C4.5 basically assesses the error rates of the tree and its subtrees using the set of training samples. If the real error of a subtree is larger than the upper confidence limit of error distribution, the subtree will be replaced by a leaf. When the pruning process is accomplished, a decision tree is finally built to make predictions. As a classic algorithm of decision tree, C4.5 is able to handle data with discrete attributes or even nonnumerical attributes. Therefore, we chose C4.5 algorithm to produce classification models based on our depression dataset.

For the methods described above, many implementations have been released over the Internet. Among them, we chose WEKA, a well-known data mining tool, to perform classification tests [21]. We utilized “local TAN” to search the structure of Bayes network and took advantage of package “libsvm” to learn SVM models [22] and employed classifier “J48” (a java implementation of C4.5) to build decision trees for syndrome classification. Additionally, to avoid biases in separating training and testing sets, we evaluated the classification performance for each syndrome by repeating 5-fold cross-validation for 10 times. Thus, the depression dataset could be fully tested by 4 kinds of methods.

### 2.3. Symptom Selection

Since classification models were unable to uncover the pattern of syndrome, we should investigate the interacting profile of symptoms for a given syndrome. For this purpose, key symptoms should be selected for 7 syndromes of depression in the first place. Although lots of methods could accomplish the task of feature selection [23], we here employed Fisher's score to select key symptoms on account of its simplicity and accuracy. Fisher's score, derived from the criterion of Fisher's linear discriminant analysis, could measure the ability of an individual feature to correctly classify the samples [24]. Given a data set {*x*_*j*_, *y*_*j*_}_*j*=1_^*n*^, *y*_*j*_ ∈ {1,2,…, *c*}. Let *μ*_*ir*_ and *σ*_*ir*_^2^ be the mean and variance of class *i*, *i* = 1,2,…, *c*, corresponding to the *r*th feature. Let *μ*_*r*_ denote the mean of the whole data set for the *r*th feature. So, the Fisher's score of feature *F*_*r*_ is defined as follows [25]:

(4) $$\begin{array}{c} S\left(F_{r}\right)=\frac{\sum_{i=1}^{c}P_{i}{\left(\mu_{ir}-\mu_{r}\right)}^{2}}{\sum_{i=1}^{c}P_{i}\sigma_{ir}^{2}}, \end{array}$$

where *P*_*i*_ is the prior probability of class *i*. According to (4), a feature would have a large Fisher's score if it has a small within-class variance and a large between-class variance given a class variable. Consequently, symptoms that are crucial to classify a given syndrome usually have large Fisher's scores. To sum up, symptoms with large Fisher's scores are highly related to corresponding syndrome in the sense of statistics and may also play an essential role in syndrome differentiation in practice.

### 2.4. Symptom Network

After key symptoms for each syndrome were selected, the underrated interacting profile of symptoms could be particularly investigated. We employed a network-based technique to explore and analyze the pattern of syndromes at symptomatic level. We constructed 8 symptom networks: one for depression and the others for 7 syndromes in depression. Through systematic investigation of these symptom networks, we could discover certain characteristics underlying depression and its syndromes.

We employed weighted mutual information (wMI) to estimate the dependence between two symptoms under a certain syndrome. Before computing wMIs for symptom pairs, we should determine the dataset for network construction with regard to a syndrome. As a matter of fact, we just took advantage of a part of depression dataset, named positive set, to construct symptom network for a given syndrome. The positive set of a syndrome is comprised of all cases belonging to that syndrome, that is, all samples with label “1” of that syndrome. For example, BD positive set includes all cases with BD syndrome labeled “1.” Similarly, the positive set of depression is actually the whole depression dataset. Then, based on the positive set of depression or a given syndrome, we could measure the correlation between any two symptom features using weighted mutual information (wMI) [26]. The wMI for symptoms *X* and *Y* can be calculated as follows:

(5) $$\begin{array}{c} \text{wMI}\left(X;Y\right)=\underset{x\in \Omega_{X}}{\sum }\;\underset{y\in \Omega_{Y}}{\sum }w\left(x,y\right)P\left(x,y\right)\log\frac{P\left(x,y\right)}{P\left(x\right)P\left(y\right)}, \end{array}$$

where *Ω*_*X*_ and *Ω*_*Y*_ are the ranges of symptoms *X* and *Y*, respectively; *w*(*x*, *y*) is a weight function concerning *X* and *Y*. We used a weight matrix to define the weight function.

Table 2 specifies weights in terms of different risk degrees of symptoms *X* and *Y*. By using such a weight function in (5), we intuitively reduced the contribution of undesired correlations (e.g., *X* = 0 and *Y* = 3) to the final wMI of *X* and *Y*. When the wMIs of all symptom pairs were computed, a threshold *θ* was set to eliminate trivial symptom interactions. Only symptom pairs with wMIs equal to or larger than *θ* were selected and considered to be correlative. On the basis of these selected symptom interactions, a symptom network could be eventually constructed for depression or a given syndrome.

### 2.5. Network Analysis

As a significant model for complex systems, complex network holds a lot of nontrivial features, such as a heavy tail in the degree distribution, a high clustering coefficient, assortativity or disassortativity among vertices, community structure, and hierarchical structure [27, 28]. These nontrivial features characterize real-world networks in different aspects. Combined with domain knowledge, some remarkable results would be discovered by analyzing the characteristics of complex networks. Consequently, we employed Cytoscape (v2.8.3) and python package networkx (v1.6) to investigate the intrinsic properties of symptom networks [29].

Beside basic topological characteristics of symptom networks, we also investigated the dynamic changes of symptom interactions under a given syndrome. We identified the dynamic symptom interactions using a “self-contained” permutation test. First, the labels of a given syndrome in depression dataset were randomly shuffled. Second, a positive set was generated for the syndrome from the shuffled dataset and then a symptom network (named random network) was constructed based on this positive set. Third, for an edge in the original network, we compared the wMI in random network to its original wMI. Fourth, repeat previous three steps for *N* times and compute *P* values for all symptom interactions in the original network. The *P* value of an interaction could be calculated as follows:

(6) $$\begin{array}{c} P_{g}\left(e_{uv}\right)=\frac{n_{g}\left(e_{uv}\right)}{N},\\ P_{l}\left(e_{uv}\right)=1-P_{g}\left(e_{uv}\right)=\frac{n_{l}\left(e_{uv}\right)}{N}, \end{array}$$

where *P*_*g*_(*e*_*uv*_) is the *P* value of the wMI of edge (*u*, *v*) larger than in random; that is, if *P*_*g*_(*e*_*uv*_) is lower than a small threshold, then wMI of edge (*u*, *v*) is believed to be larger than in random, indicating that edge (*u*, *v*) became more correlated under the syndrome; *P*_*l*_(*e*_*uv*_) is the *P* value of the wMI of edge (*u*, *v*) less than in random; *n*_*g*_(*e*_*uv*_) was the number of iterations with equal or greater wMI of edge (*u*, *v*) in random network than the original; and *n*_*l*_(*e*_*uv*_) = *N* − *n*_*g*_(*e*_*uv*_). Then symptom interactions with significant *P*_*g*_(*e*_*uv*_) or *P*_*l*_(*e*_*uv*_) were considered to be dynamically changed.

Typically, the dynamic interactions together with key symptoms could represent the pattern of syndrome in depression. Key symptoms played an essential role in differentiating syndromes and dynamic interactions uncovered the difference of symptom pairs under different syndromes. Therefore, symptom networks could facilitate our understanding of syndromes and improve the accuracy of syndrome differentiation in depression.

## 3. Results

### 3.1. Syndrome Classification

**Figure 1:**
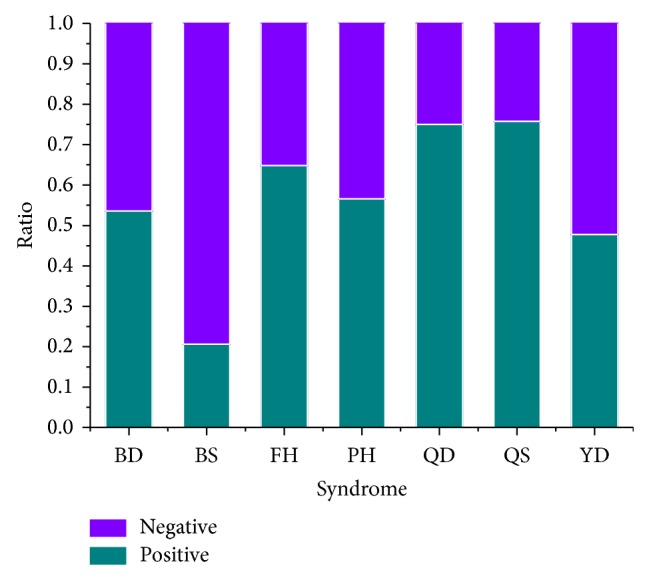
Distribution of syndromes in depression. The “positive” refers to cases that belong to a certain syndrome; “negative” is cases without that syndrome.

When the diagnosis information of depression patients were collected and preprocessed, we explored the distribution of syndromes in depression (Figure 1). On the basis of depression dataset, we investigated the ratios of positive samples and negative samples corresponding to every syndrome in depression. Obviously, Qi stagnation (QS), Qi deficiency (QD), and fire hot (FH) syndrome were enriched in the whole dataset. It implied that patients suffering from depression were likely to have these syndromes. Namely, doctors aiming to treat depression by TCM therapies could endeavor to release the risk of these syndromes, such as Xiao Yao San decoction and acupuncture [30, 31]. It suggested that although depression is a kind of affective disorder, therapies to relieve different syndromes have been a complementary treatment to the psychological counseling for depression patients [30]. From this point of view, syndrome differentiation becomes important and necessary to treat depression using TCM therapies.

**Figure 2:**
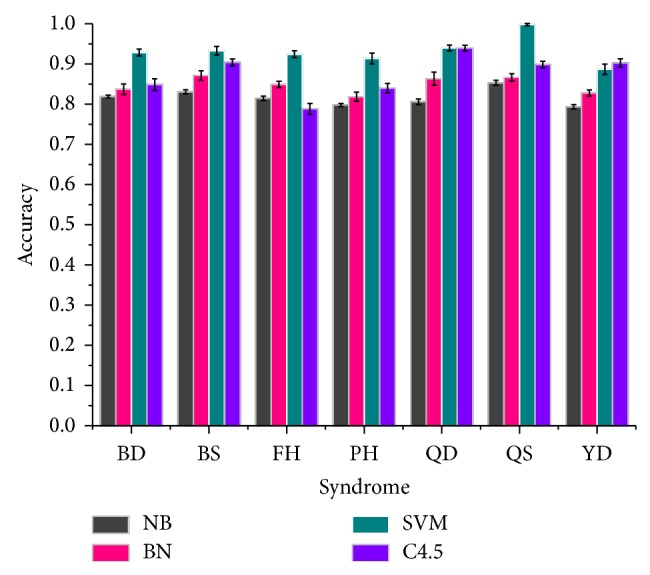
Performance of classification on 7 syndromes using all 36 symptom features. NB stands for naïve Bayes method and BN represents Bayes network classifier. Accuracy is the average of accuracies of 5-fold cross-validation over 10 trials for classification of every syndrome. The error bar at the top represents the standard deviation of 10 trials for each method.

Next we learned computational models to classify syndromes in depression and then evaluated the outcome of classification test [32]. For each syndrome, we repeated 5-fold cross-validation for 10 times using 4 different methods. The performance of every method was measured by average accuracy, that is, the average ratio of correctly classified samples over 10 trials.  Figure 2 explicitly showed performance of four classification methods in 7 syndromes. In general, SVM achieved the best performance among the 4 methods (*t* test; *P* value 2.2*e* − 16 for BD; *P* value 1.0*e* − 11 for BS; *P* value 2.2*e* − 16 for FH; *P* value 4.2*e* − 14 for PH; *P* value 9.4*e* − 8 for QD; *P* value 2.2*e* − 16 for QS) except for YD syndrome. C4.5 had comparable performance with SVM in identifying QD and YD syndrome but became the worst in FH (*P* value 1.9*e* − 9). Naïve Bayes and Bayes network usually achieved lower accuracies than SVM and C4.5 in classification of 7 syndromes, but C4.5 in FH identification was an exception. Overall, the accuracy of four methods in 7 syndromes was mainly distributed around 0.85. In other words, this dataset was statistically credible although there lacked diagnostic information of tongue and pulse for each patient in the original medical records. Most importantly, Bayes network outperformed naïve Bayes more or less in any classification test of 7 syndromes (*P* value 9.8*e* − 4 for BD; *P* value 2.6*e* − 7 for BS; *P* value 4.8*e* − 9 for FH; *P* value 1.7*e* − 4 for PH; *P* value 2.4*e* − 7 for QD; *P* value 1.3*e* − 3 for QS; *P* value 3.1*e* − 9 for YD) according to Figure 2. It implied that the hypothesis of conditional independence underlying naïve Bayes was not true as stated in (2). Namely, 36 symptoms were not fully uncorrelated under any syndrome in depression. Therefore, the exploration of symptom interaction is of great value to facilitate our understanding of the pattern of syndrome in depression.

### 3.2. Key Symptoms in Depression

Although depression patients were inquired about 36 types of physical signs, TCM doctors always differentiated syndromes based on a small group of key symptoms. Namely, a combination of several key symptoms could represent the general pattern of a certain syndrome in depression. We here employed Fisher's score to discover the symptom combinations corresponding to 7 syndromes. For each syndrome, 12 symptoms with largest Fisher's scores were selected as shown in Table 3. Obviously, top 3 key symptoms were unique in each syndrome. For example, none of PS5, PS6, or PS8 of QD syndrome was ranked in top 3 of other syndromes. This indicated that 7 syndromes in depression were specific to a certain extent from symptomatic perspective. On the other hand, significant cooccurrence of key symptoms was observed between different syndromes. For example, PS27 played an important role in BD, PH, and QS identification. It suggested that 7 syndromes may correlate with one another and cooccur in depression patients. Therefore, the specificity of 7 syndromes and the correlation between them were both uncovered through their key symptoms.

**Figure 3:**
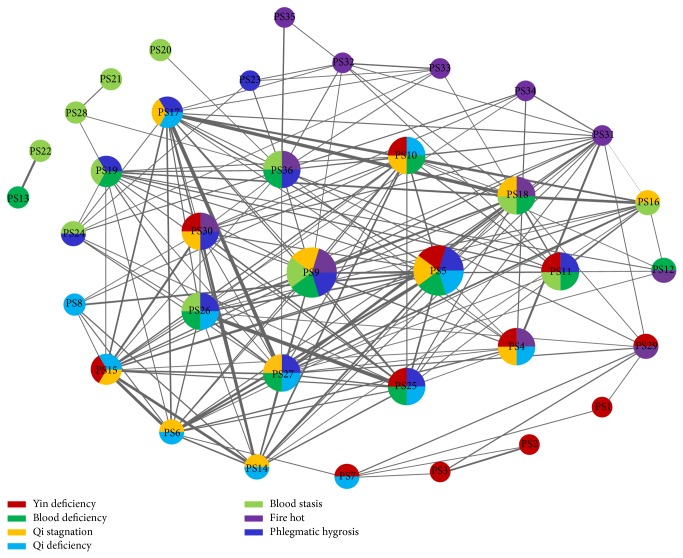
Depression network. An edge indicates the relation between two connected symptoms. All edges are weighted by wMIs of corresponding symptom pairs. A thick edge represents strong relation between two nodes. All edge weights (wMIs) are not less than 0.1. The color of a node indicates the syndrome that symptoms are highly related to. Only top 12 key symptoms of each syndrome are marked by colors. A multicolor node implies that the symptom plays a crucial role in classifying different syndromes. For example, PS27 is of vital importance to differentiate between BD, PH, QD, and QS syndrome.

Besides symptom combinations, we further studied the correlation between syndromes in depression using a network-based technique. So a symptom network was constructed for depression and key symptoms of each syndrome were mapped into this depression network (Figure 3). The nodes were 36 symptoms and the edges were significant interactions between symptoms. We selected edges using wMIs of symptom pairs, and the threshold for wMI was set to *θ* = 0.1. As shown in Figure 3, 7 syndromes were grouped by different colors of nodes. In fact, we employed top 12 key symptoms as a brief representation of each syndrome (Table 3). It was obvious that more than half of 36 symptoms highly correlated with two or more syndromes. In particular, PS5 and PS9 were both related to 5 syndromes, which implied that exhaustion and dry eye may be the common physical signs of depression and could be used to forecast depression. In addition, 6 syndromes except QS correlated with at least one specific symptom (monocolor nodes). The number of specific symptoms was 5 for FH, 4 for BS, 3 for YD, and 1 for BD, QD, and FH. Note that the distribution of multicolor and monocolor nodes depended on the number of key symptoms we selected for each syndrome. Even so, we could find that FH, BS, and YD were relatively separated from other syndromes. Moreover, PS17, PS18, and PS27 were connected by thick edges in Figure 3. In fact, they were all evident symptoms about obstruction in chest or stomach. Similarly, PS25 and PS26 were both related to dizziness. It suggested that certain symptoms were inherently close or similar.

**Figure 4:**
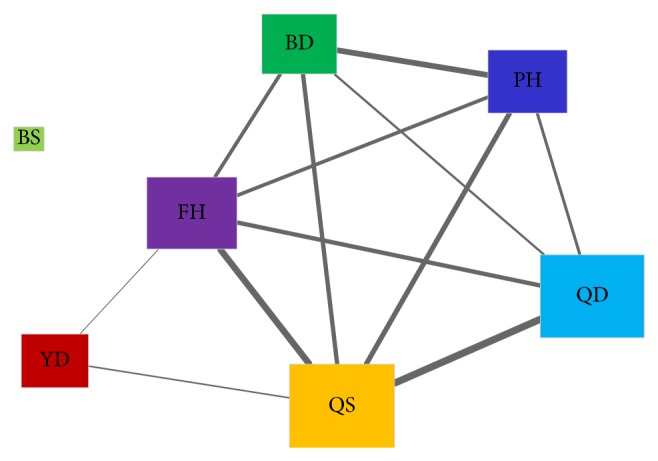
Syndrome network in depression. An edge represents an interaction between two syndromes. All edges are weighted by Tanimoto similarities of syndrome pairs. The node size is proportional to the probability of each syndrome occurring in depression patients.

Although the depression network could partly uncover the intrinsic relations between different syndromes, we further took advantage of clinical data to explore the characteristics of syndromes in depression. We computed the Tanimoto similarity for all syndrome pairs. The Tanimoto similarity between syndromes *S*_1_ and *S*_2_ was calculated as follows:

(7) $$\begin{array}{c} \text{sim}\left(S_{1},S_{2}\right)=\frac{c}{a+b-c}, \end{array}$$

where *c* is the number of patients having both syndromes *S*_1_ and *S*_2_; *a* is the number of cases belonging to *S*_1_; *b* is the number of cases belonging to *S*_2_. Only interactions with similarities equal to or larger than 0.5 were selected to construct a syndrome network. According to Figure 4, QS and QD tended to cooccur in depression patients (Fisher's exact test; *P* value 2.4*e* − 7), so did FH and QS (*P* value 1.4*e* − 6). BS and YD patients were unlikely to have other syndromes in depression, as discussed above. Furthermore, BD, FH, QS, QD, and PH formed a full-connected subnetwork, implying that these syndromes may tend to occur in patients with each other instead of individually. To sum up, five syndromes in depression, BD, FH, QS, QD, and PH, highly correlated with one another and the remaining two, BS and YD, were relatively separated from other syndromes.

### 3.3. Symptom Network for 7 Syndromes

Since the relations between symptoms made contribution to syndrome classification in depression, we particularly investigated the characteristics of syndromes from the perspective of symptom network. We employed top 12 key symptoms as a brief representation of each syndrome (Table 3) and constructed networks based on these symptoms. The threshold for wMIs was set to *θ* = 0.1, which meant that only symptom pairs with wMIs no less than 0.1 were selected to construct a network. In the end, symptom networks for 7 syndromes were constructed following the procedure described above.

We primarily analyzed the topological characteristics of symptom networks for depression and 7 syndromes. Some important properties of symptom networks were listed in Table 4. Because 5 of the 8 symptom networks were not fully connected, the comparison of average shortest path, radius, and diameter would not uncover informative findings. We paid attention to other properties instead. According to Table 4, we found that BS, QD, and QS network had comparable clustering coefficients to depression network. It implied that symptoms in these networks tended to correlate with others. This was confirmed by large densities of BS, QD, and QS network. The small density of depression network resulted from relatively large number of nodes. In addition, BD, FH, QD, and YD network had two connected components. It suggested that a syndrome may cause uncorrelated physical signs in depression patients, such as PS12 in FH network and PS2 in YD network.

**Figure 5:**
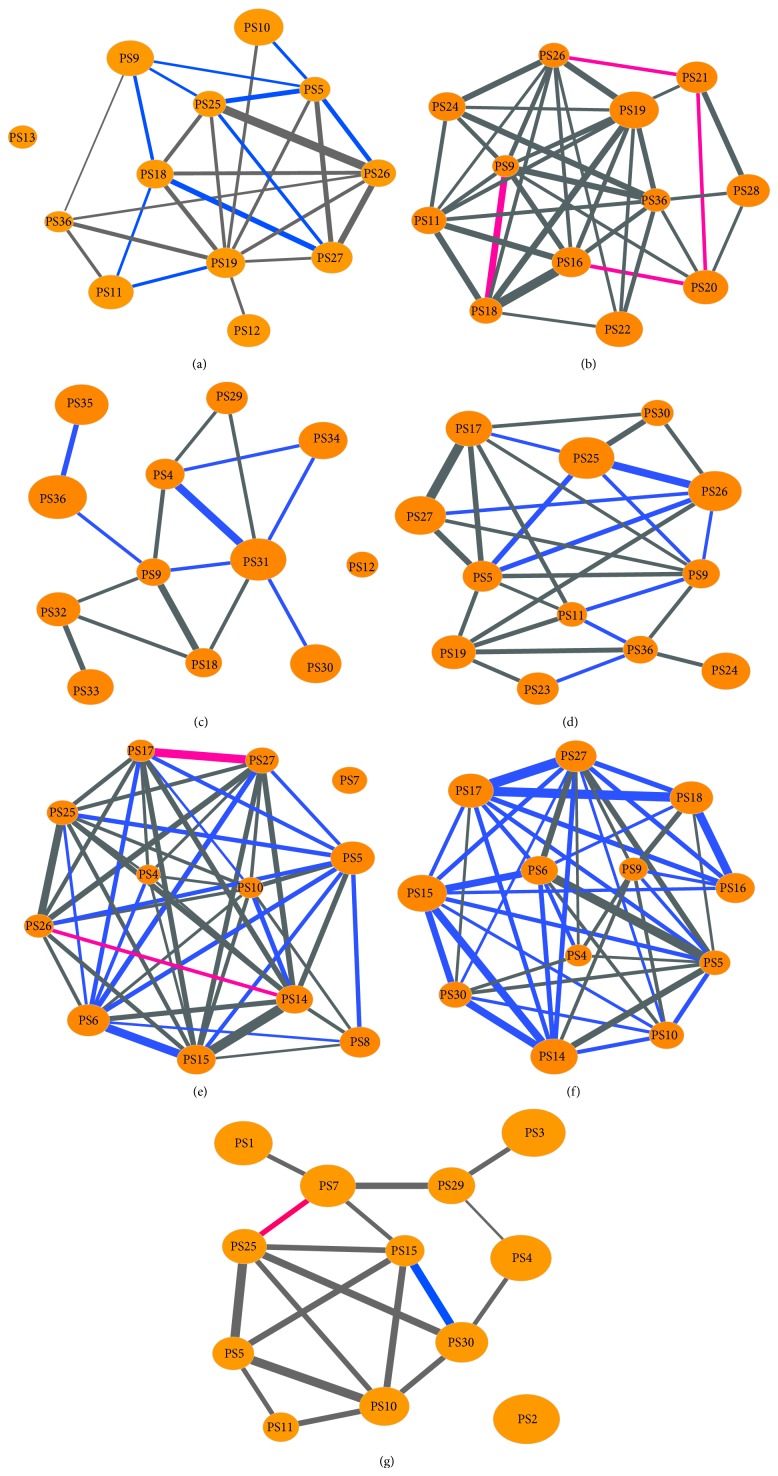
Symptom networks for 7 syndromes in depression. (a) BD (blood deficiency) symptom network. (b) BS (blood stasis) network. (c) FH (fire hot) network. (d) PH (phlegmatic hygrosis) network. (e) QD (Qi deficiency) network. (f) QS (Qi stagnation) network. (g) YD (Yin deficiency) network. Only top 12 key symptoms for each syndrome are employed to construct a corresponding symptom network. All edges are weighted by wMIs of symptom pairs that are not less than 0.1. The red edges indicate that wMIs of corresponding symptom interactions are greater than in random (*P*_*g*_ < 0.1), while the blue edges represent symptom interactions with less wMIs than in random (*P*_*l*_ < 0.1); the remaining edges (gray edges) are not statistically significant. A large node implies a high rank for classifying a certain syndrome.

Besides the topological properties, we further investigated the specific characteristics of symptom networks including dynamic changes of symptom interactions. Symptom networks for 7 syndromes were exhibited in Figure 5. Generally, highly ranked symptoms (nodes with large size) for BD, FH, and YD syndrome had few neighbors and low degrees. However, such tendency was not evident in BS, PH, QD, and QS network. On the other hand, a symptom usually had diverse topological properties in different symptom networks. For example, PS36 had a large degree in BS network, while it only connected two other nodes in FH network. There were also several symptoms playing “hub” role in multiple symptom networks, such as PS5 and PS9. In fact, such symptoms could be used to determine whether a patient suffered from depression or not.

Since certain symptoms were inherently close or similar, we employed a technique of permutation test to identify the dynamic changes of symptom interactions. According to the procedure described in Section 2, we here repeated the permutation test for 10000 times and set the cutoff for *P* values to 0.1. Namely, all edges with *P*_*g*_ or *P*_*l*_ less than 0.1 were considered to be dynamic symptom interactions (Figure 5). We further distinguished dynamic interactions in terms of *P* values. We defined edges with *P*_*g*_ less than 0.1 as increased interactions (red edges in Figure 5); and edges with *P*_*l*_ less than 0.1 were decreased interactions (blue edges). The number of decreased and increased interactions of each symptom network was summarized in Table 4. Obviously, decreased interactions were far more than increased interactions. The increased interactions were discovered in BS, QD, and YD network. Most symptom networks just had one type of dynamic interactions. For example, BS network had 4 increased interactions and QS network had 29 decreased interactions. Only QD and YD network had both decreased and increased interactions. Although the number of dynamic interactions varied in terms of different syndromes, we should further explore the symptom networks to discover the underlying pattern of syndromes.

To uncover the difference in symptom networks, we explored the dynamic interactions identified by permutation test. A dynamic interaction usually implied certain knowledge about syndrome. For example, symptom pair PS25 and PS26 was less correlated in PH network than at random (*P*_*l*_ = 0.0134). It implied that PH patients were more unlikely to have symptoms PS25 and PS26 at the same time than other depression patients. Similarly, BS patients probably had symptoms PS9 and PS18 simultaneously (*P*_*g*_ = 0.0946). Moreover, we found that symptom pair PS17 and PS27 was an increased interaction in QD network (*P*_*g*_ = 0.041) but a decreased interaction in QS network (*P*_*l*_ = 0.0161). It implied that PS17 and PS27 tended to cooccur in QD patients but not in QS patients regardless of its inherent similarity. The pair PS17 and PS27 was also present in PH network but not in a significant dynamic interaction. Consequently, we could partly learn the pattern of PH, QD, and QS syndrome based on the difference between PS17 and PS27 in their symptom networks. Therefore, the pattern of syndrome in depression could be uncovered based on the difference of symptom networks.

## 4. Conclusion

In this paper, we explored the pattern of syndrome based on the difference of symptom network in depression. We firstly performed classification test for 7 syndromes in depression. Then we selected key symptoms using Fisher's score and constructed symptom networks for depression and 7 syndromes. Finally, we analyzed the pattern of syndrome through dynamic interactions identified in each symptom network. Although this workflow could uncover the pattern of syndrome from clinical diagnosis data, several inevitable disadvantages affect the final conclusion we draw. The first is the subjectivity inherent in TCM diagnosis and treatment. As stated before, the determination of the risk degree of every symptom may be incorrect. What a patient feels is difficult to measure and standardize. Sometimes, patients may not inform the doctor of some personal conditions. Hence, there is always noise or redundancy in the diagnosis data. The second disadvantage comes from the similarity of symptoms. Though the symptoms are explicitly described, the similarity between certain symptoms would definitely affect the final results of network analysis. For an instance, PS17 and PS18 are almost the same except for a little difference, and PS13 and PS22 have similar definitions both specified to females. These similar symptoms may confuse patients when inquiring the physical sign of a disorder and then bring bias to raw data for experiment. Although some technique could reduce such bias, the reliability of processed data is still doubtable. The third is the small quantity of depression dataset. Since the bias is inevitable in TCM diagnosis dataset, large proportion of medical records could greatly reduce the bias. On the contrary, a small data set usually leads to an unreliable result. Thus, computational models learned from a small dataset need to be tested and validated carefully.

After the diagnosis data were collected, we learned classification models for each syndrome rather than a single multilabel classifier. Actually, the way we trained models for syndrome differentiation is a kind of “problem transformation methods” to address multilabel classification problem [33]. The fact that a single multilabel classifier for 7 syndromes was not adopted mainly concerns two reasons. Firstly, a single model may achieve good performance for one or more class labels but becomes worse for others. As shown in Figure 2, SVM is the best for 6 syndromes but not as good as C4.5 for YD (Yin deficiency) syndrome. Secondly, the number of cases belonging to BS (blood stasis) is quite small (Figure 1) and BS does not evidently correlate with other syndromes (Figure 4). Some multilabel methods may not solve such small sample-size problem with other rich labels (FH, QD, QS, and so on). Thus, a classifier for each syndrome is a rational choice for syndrome differentiation in depression. In the end, the workflow could uncover the pattern of syndrome using scaled diagnosis data. We could not only determine the symptom combinations for 7 syndromes using Fisher's score but also investigate the changes of symptom interactions in terms of different syndromes. A symptom network rather than a group of symptoms is exploited to represent the pattern of a given syndrome in depression. Hence, this approach provides new insight into the exploration of syndrome difference and essence in a computational manner. The knowledge underlying dynamic interactions of symptom network for a syndrome could facilitate our understanding of the pattern of syndrome and further improve the accuracy of syndrome differentiation.

## Figures and Tables

**Table 1:** Description of 36 symptoms related to depression.

Symptom	Description
PS1	Feeling hot in palms or soles but cool in body
PS2	Sweating when sleeping but not after waking up
PS3	Feeling hot off and on, especially in midnight or at afternoon
PS4	Feeling dry in mouth and throat lately
PS5	Feeling limply and exhausted
PS6	Feeling short of breath, especially after sports
PS7	Sweating a lot by day, especially after sports
PS8	Feeling tired in spirit, lazy, and wordless
PS9	Having blurred vision and feeling dry in eyes
PS10	Feeling nervous and having super abundance of dreams
PS11	Having hair thin and dry and easily falling out
PS12	Ever feeling numb in your hands, feet, and body
PS13	(Female) the menses being little in quantity and light in color
PS14	Feeling depressed
PS15	Feeling tight in chest and like long out of breath
PS16	Feeling swelling pain in lateral thorax
PS17	Feeling obstructive in epigastrium and indigestive after taking food
PS18	Feeling swelling pain in stomach even in hypogastrium or costal region
PS19	Feeling pain like prodding in a certain part of the body
PS20	Having suggillation in your skins
PS21	Having a sclerotic lump detected in body
PS22	(Female) the menses being dark purple in color or with some blood clots
PS23	Coughing up dense phlegm
PS24	Feeling nauseous and wanting to vomit
PS25	Feeling heavy and dazed in head
PS26	Feeling dizzy and fainty
PS27	Feeling obstructive in chest or gastric cavity
PS28	Having smooth lumps in some organ of your body
PS29	Feeling febrile in your body, especially in chest and stomach
PS30	Being impatient and irritable
PS31	Ever feeling bitter taste in mouth
PS32	Having mouth or tongue ulcerated
PS33	Having gingiva gall
PS34	Usually feeling thirsty and wanting to drink something cold
PS35	Having constipation
PS36	Having urine little in quantity and yellow in color

**Table 2:** 

	*Y* = 0	*Y* = 1	*Y* = 2	*Y* = 3
*X* = 0	1.0	0.8	0.6	0.4
*X* = 1	0.8	1.0	0.8	0.6
*X* = 2	0.6	0.8	1.0	0.8
*X* = 3	0.4	0.6	0.8	1.0

**Table 3:** Key symptoms for 7 syndromes in depression.

Rank	BD	BS	FH	PH	QD	QS	YD
1	PS10	PS19	PS36	PS25	PS5	PS15	PS2
2	PS9	PS22	PS31	PS26	PS6	PS14	PS3
3	PS11	PS20	PS35	PS27	PS8	PS17	PS4
4	PS12	PS28	PS30	PS24	PS15	PS18	PS1
5	PS27	PS21	PS34	PS17	PS14	PS27	PS7
6	PS19	PS16	PS33	PS19	PS7	PS16	PS30
7	PS18	PS24	PS32	PS23	PS27	PS6	PS10
8	PS26	PS11	PS29	PS5	PS25	PS10	PS29
9	PS25	PS18	PS4	PS9	PS26	PS30	PS25
10	PS5	PS26	PS18	PS36	PS17	PS5	PS5
11	PS13	PS36	PS9	PS30	PS10	PS9	PS15
12	PS36	PS9	PS12	PS11	PS4	PS4	PS11

**Table 4:** Topological characteristics of symptom networks for depression and 7 syndromes. NN—number of nodes; NE—number of edges; NCC—number of connected components; CC—clustering coefficient; ND—network density; NDI—number of decreased interactions (edges with less wMI than random); NII—number of increased interactions (edges with greater wMI than random).

Network	NN	NE	NCC	CC	ND	NDI	NII
Depression	36	176	2	0.621	0.279	—	—
BD	12	26	2	0.505	0.394	10	0
BS	12	37	1	0.666	0.561	0	4
FH	12	15	2	0.339	0.227	7	0
PH	12	28	1	0.550	0.424	10	0
QD	12	48	2	0.842	0.727	15	2
QS	12	45	1	0.707	0.682	29	0
YD	12	18	2	0.344	0.273	1	1
